# Dr. Andrew H. Smith

**Published:** 1910-05

**Authors:** 


Dr. Andrew H. Smith, of New York, one of the best known medical practitioners in that city, died at his home April 8, 1910, of arteriosclerosis and myocarditis, aged 72 years. He served as a medical officer in the Civil War, remaining in the army until 1867. He was president of the New York Academy of Medicine, 1903-1904, and vice-president of the New York Medical School and Hospital since 1895. He was a companion of the military order of the Loyal Legion of the United States, his insignia being No. 883, one of the earlier ones.

